# Hospital and Institutional News

**Published:** 1921-02-05

**Authors:** 


### HOSPITAL AND INSTITUTIONAL NEWS.

### THE COMMITTEE OF INQUIRY.

The Committee of Inquiry, under the Chairmanship of Viscount Cave, held its first full meeting on January 26. The first witnesses were Sir Arthur Stanley, Chairman of the British Red Cross Society and treasurer of St. Thomas's Hospital; Sir Napier Burnett, Director of Hospital Services under the British Red Cross; and Viscount Knutsford and Mr. E. W. Morris, of the London Hospital. It is requested that hospital chairmen and any organisations desirous of giving evidence will communicate at once with the Secretary of the Committee, Mr. L. G. Brock, C.B., at the Ministry of Health. The two completing members of the Committee are Lord Linlithgow, nominated by the Secretary for Scotland, and Sir W. B. Peat.

### THE PRINCE OF WALES AT BRIGHTON.

The programme of the Prince of Wales's visit to Brighton on Tuesday has been abundantly described in the newspapers, but our readers may welcome a personal impression, which the good fortune of being present enables us to give. The children almost universally were given the front place. At many points of the route, indeed, the policemen themselves stood behind the children! This goodfellowship permeated the crowd, which was, even for an English crowd, exceptionally good-tempered, easily managed, and considerate to itself. As we moved along it friendly confidences were being exchanged. One good woman lamented that the Prince was not, throughout the entire day, in uniform; another remarked that she saw the same people near her in the afternoon as she had found at a distant part of the route in the morning. A small boy who found the waiting a little long and began to give vent to impatience was quieted by an enormous policeman, who said: "If you do not keep quiet, sonny, I shall stand in front of you when the Prince comes." The ubiquitous nurse lent a touch of distinction here and there. Hers was the uniform most apparent in the streets, and she, like her fellow-citizens, was mostly disinterested, with a patient for whose pleasure she was planning the best point of vantage from which to see the Prince of Wales.

### THE BIRMINGHAM HOSPITAL JOURNAL.

Among the interesting periodicals devoted to institutional affairs is *The G.H.B.*, or the journal of the General Hospital, Birmingham. Whatever may be thought of the controversy between the Birmingham General Hospital League, of which this is the official monthly organ, and its sister organisations, the magazine makes interesting reading. A leading feature of the January number is an article signed "H. B. S.," entitled "A Justification." The writer regrets the recent "misunderstanding," as must everyone on either side. He affirms, however, that the state of the General Hospital is "desperate," that it needs relief "*at once*," and that the League alone can provide this succour. "A promise of help in twelve months' time is not sufficient." On this ground the writer argues that the League "has been started in a genuine attempt to save the voluntary system," as well as the General Hospital, and that experience has shown that, since maintenance charges are very unpopular in Birmingham, the League is "the best means of raising money immediately." He asserts, further, that the workers' representatives from their various hospital fund committees have discussed the question with judgment, and asks, perhaps a little combatively: "Is the right of giving their own money to whatever charity they choose to be denied to these men?" Of course not, but a *suaviter in modo* would have avoided the suggestion, however inadvertent, that these donors prefer one charity to another. Some competition is unavoidable in a voluntary system. Whether it braces or embitters depends upon those who guide it. The argument, however, is intended not offensively, though it might have been more tactfully expressed, against other institutions, but against a central distributing agency, which, it is affirmed, converts committees into "animated collecting-boxes." Well, well, we sympathise much with the argument, which has many supporters, not only in Birmingham. But the distributing agency works equally well in other places—for instance, in London. The proper plan is to see if both methods cannot be combined, and if the spirit of the League can make it plain that it welcomes co-operation no less than competition everyone will be in its debt.

### THE APPEAL FOR NEWCASTLE'S COLLEGES.

Although something like half a million pounds is the sum required to put the two University Colleges on a firm financial basis, yet the response already received to their appeal has put renewed heart into the authorities tackling the tremendous task. We understand that, as we go to press, the total receipts amount to well over £90,000. Locally the proceedings are described as a whirlwind campaign, and the methods employed certainly appear likely to leave no possible source of financial aid untapped. The economic situation in Newcastle is such that it is absolutely necessary to maintain a first-class system of technical and scientific education in the city, and, apart from expressing our sympathies with the authorities in a very efficient management of their own affairs, we are glad that their propaganda is explaining at the same time, and to the whole country, the value of University education. With regard to the Newcastle College of Medicine, which can already claim to have given the country many a first-class professional man, it was recalled in a speech by Professor Grey Turner that it had its beginning in the city less than ninety years ago, and that in a little room in Bell's Court, off Pilgrim Street.

### THE ST. BARTHOLOMEW'S PEACE APPEAL.

Few people realise the very heavy work which fell upon the shoulders of Mr. A. F. Shepherd, the originator and organiser of the Peace Commemoration Appeal of St. Bartholomew's Hospital, which resulted in the receipt of £132,000 by the hospital. At the general court of Governors held on January 29, a resolution was passed expressing sincere thanks to Mr. A. F. Shepherd for his services. The Governors, in this resolution, mentioned that it is anticipated that further benefactions will accrue in consequence of the widespread publicity given to the work and needs of the institution.

### PRESENTATION TO HOSPITAL SECRETARY.

At a large and representative gathering of members of the Board of Management, the medical staff and governors and subscribers of St. Mary's Hospital a parting gift to Mr. Thomas Ryan, the late Secretary, on his retirement after thirty-three years' service, in the shape of a substantial cheque was made. The presentation was made by Mr. Arthur R. Prideaux, who spoke in warm terms of Mr. Ryan's distinguished services to the hospital, which during his long period of service has made great advances in efficiency and material prosperity.

### THE HOSPITAL SATURDAY FUND.

We made reference last week in a leading article to the receipts of the Hospital Saturday Fund, and mentioned £30,000 as the approximate increase of the income for 1920 over that for the previous year. We find, with pleasure, that we have understated the amount. The true figures are £105,987 for 1920 and £74,601 for 1919. The collection represents the largest amount collected in one year by the Fund, which has since its inception distributed one million pounds amongst the hospitals of London. The King, who is Patron of the Fund, has congratulated the 20,000 honorary workers and the officers on the gratifying results of an efficient organisation.

### THE ANTHRAX DANGER.

The official advice just published in connection with the possible dangers of anthrax infection in this country recall the amusing discussions appearing a few months ago upon the efficiency of suggested disinfecting methods for shaving-brushes—efficient not only for destruction of the anthrax spore, but of the offending brush as well. There is no doubt, however, that before the importation of Japanese brushes was prohibited sufficient entered the country to leave, even to-day, in the hands of the retailers numbers of Japanese brushes, which must be regarded as dangerous. Every effort is being made to locate them, and meanwhile it is advised that the best practicable method of partially disinfecting the brushes is (*a*) to wash them thoroughly with soap in warm water containing a little washing soda, and to allow them to stand for half an hour in water containing this soda; (*b*) to place them in a warm 2½ per cent. solution of formaldehyde for half an hour; and (*c*) allow them to dry. Even this does not, of course, deal with spores embedded in the handle of the brush, and Sir George Newman's circular is more valuable in drawing attention to the latest experiences in treatment. Excision of the pustule does not now appear to be the first consideration. In general the best treatment is physiological rest, combined with intravenous injection of anti-anthrax serum, 60-80 c.c. on the first day and 60 c.c. on the second, according to the patient's reaction. The superiority of the serum appears to be supported strongly in figures supplied by the Medical Inspector of Factories.

### THE REPORT ON INFLUENZA.

The report on the Pandemic of Influenza, 1918-19, has been issued by the Ministry of Health, and is obtainable from H.M. Stationery Office. Part I., which deals with Great Britain and Ireland, includes the history of influenza in England from 1658-1911, the clinical features of the 1918-19 epidemics, together with an analytical discussion of the bacteriology, infectivity, etc., and also demonstrations of the relation between meteorological conditions, death-rates, domestic overcrowding, and prophylaxis. Part II. of the report relates to influenza in foreign countries, and Part III. contains reports on a number of local investigations undertaken by medical officers of health and others. At best, however, the problem is but partially solved. It remains, indeed, but a portion of a still wider problem —that of the health and happiness of mankind.

### FOOD POISONING CASES.

A variety of so-called food-poisoning cases have been lately met with in London practice, and the popular theories as to their origin are chiefly remarkable for their improbability; but the Ministry of Health, in a recent circular to medical officers of health, wisely requests that when they have reason to suspect any death or illness to be due to food infection, they should promptly notify the Ministry. The real point is, however, that early notification by the attending doctor should be made, for, although under existing regulations neither the general practitioner nor the patient is obliged to report such cases to the medical officer of health, a moment's consideration will make it apparent that there is every reason, hygienic and social, for bringing them to his notice immediately. The circular (Memo. 39, Foods) indicates, besides the general procedure, the types of inquiries appropriate for outbreaks of poisoning by meat foods and possible contamination with inorganic poisons.

### MATERNITY FEES.

We note, in the correspondence columns of a Liverpool contemporary, that a medical practitioner protests with some justification against the scale of payments ordered by the Minister of Health to doctors who are called in emergency cases by midwives. As an instance, it is cited that for attendance on a midwifery case from its commencement until completion, including operative assistance with subsequent visits for ten days, if required, a fee of two guineas is allotted. Appreciating the difficulty of some of these cases and the proportion of a doctor's time they may absorb, as well as the discredit to the doctor arising from a possibly unsatisfactory result, we think that the rates of remuneration would certainly bear some degree of official reconsideration.

### DANGEROUS DRUGS ACT REGULATIONS.

DISAGREEMENT with these draft regulations (see THE HOSPITAL, January 22, p. 386) continues to be generally expressed, and a further protest has been addressed to the Home Secretary by the Secretary of the Pharmaceutical Society, stating that in the opinion of the Society's Committee appointed to deal with the matter, the Regulations as drafted cannot possibly be complied with. A series of suggestions for amendment are in preparation, and will be forwarded by the Council of the Pharmaceutical Society to the Home Secretary, but it is stated that these suggestions will embody more than "representations on details," for the Regulations appear, in the Committee's view, to have been drawn with a deliberate and ruthless disregard of the conditions surrounding the supply of the substances dealt with, and its objections to some of the Regulations are necessarily fundamental.

### DR. BUNCOMBE'S RESIGNATION.

AFTER some thirty years' service Dr. W. B. Buncombe, medical officer of Homerton and Bow institutions, has sent his resignation to the City of London Guardians on the grounds of ill-health. It was on May 20, 1890, that he became assistant medical officer to the Guardians, and he succeeded his father in the chief officership, when Dr. Buncombe, senior, resigned after having held the post for nearly fifty years. The strain of the war occasioned a serious illness, and Dr. Buncombe has requested to be relieved of his duties next March. There is no more agreeable character in institutional life than the relation of successive generations of the same family to a single institution. But we fancy that few administrative posts can be found which have been held without a break for eighty years by a doctor and his son.

### THE POSITION AT TUNBRIDGE WELLS.

"SOME people appear to consider that the position of London and provincial hospitals is exactly the same, whereas there is a great deal of difference, the position of provincial hospitals, generally speaking, being more favourable than that of, at any rate, some of the London hospitals. In the provinces there is a great spirit of local pride in the local hospital, which secretaries realise more than anyone else, as they come closely into contact with the thousands of people who are in one way or another helping." Thus Mr. J. J. Webb, Secretary of the General Hospital, Tunbridge Wells, and there is a great deal of truth in what he says. He tells us that the hospital never had a real deficit before 1919, now the bank overdraft is £4,425, the cause being the cessation of Government grants, the rise in cost, and the postponement of an appeal to avoid clashing with the local effort for a War Memorial. But in 1920 the hospital got well under way in its attempt to improve the position, with the splendid result that income from all sources was practically doubled. Annual subscriptions are about £450 more than they have ever been before, and one of the most gratifying features of the past year was the interest shown by workpeople and other classes who use the hospital. So well are things going that it is thought best at present to defer consideration of any system of payments of patients other than by voluntary gifts.

### BIRKENHEAD AND THE ANATOMY ACTS.

BOARDS of guardians are the legal custodians of the unclaimed bodies of the dead, and Dr. Alexander Macphail, of the Ministry of Health, recently urged the Birkenhead Guardians to hand over all unclaimed bodies to Liverpool University first for anatomical research, and secondly for burial. The matter next came before the Infirmary Committee of the Birkenhead Guardians, who after some discussion among themselves agreed to yield to the Ministry's request by fourteen votes to five. Mrs. Gossage, the chairman of the committee, reminded members that an unclaimed body is legally one whose relatives the Guardians fail to discover. So far as we know, no objection can be raised so long as adequate steps are taken really to discover the relatives, who are suspicious and sensitive, as can readily be understood, upon the subject.

### NEW HOSPITAL POST IN SHEFFIELD.

THE Joint Hospital Board, in the interests of all the medical charities of Sheffield, has now existed for one year, and to make it more useful an organising secretary is to begin work on January 1. Mr. Sydney Lamb has been chosen out of 150 candidates. He is not only a liveryman and a freeman of the city, where he has been engaged in business for twenty years, but also a chartered secretary, and has been the organising head of the British and Foreign Sailors' Society. On its centenary a fund of £150,000 was raised largely by his efforts. He also conducted a war savings campaign in the Navy, holding the rank of Lieutenant. The scheme resulted in a thrift organisation among British seamen. At the end of the war Mr. Lamb was appointed assistant-director of the Industrial Welfare Society, which brought him into contact with the chief employers and industries of the north. He hopes among other things to improve the organisation of workmen's contributions in Sheffield, a task in which he has everyone's good wishes.

### A NEW POST AT BEDFORD.

AT a recent meeting of the Bedford and District Workers' Hospital Fund it was resolved to appoint a full-time organising secretary at a salary of £150, with 10 per cent. on all new collections up to £1,000, when the salary will be reconsidered. Mr. J. W. Seamark, who presented the committee's report, said that the fund has made little progress since the last meeting. Seven new centres have been opened, but nine have dropped out. Much spade-work is required. The fund was started on the basis of ½d. a week. This should be raised at least to 1d. Then the new secretary will have a fair chance.

